# Trends in obesity prevalence during early pregnancy in Gran Canaria, Spain, 1993 to 2022

**DOI:** 10.3389/frph.2026.1922881

**Published:** 2026-08-26

**Authors:** Inmaculada Bautista-Castaño, Helmut Schröder, Laura Molero Sala, Alicia Inmaculada Martín Martinez, Verónica Dávila-Batista, Luis Peña Quintana, Lluis Serra-Majem

**Affiliations:** 1 Centro de Investigación Biomédica en Red Fisiopatología de la Obesidad y la Nutrición (CIBEROBN), Instituto de Salud Carlos III, Madrid, Spain; 2 Institute of Biomedical and Health Sciences Research (IUIBS), University of Las Palmas de Gran Canaria, Las Palmas de Gran Canaria, Spain; 3 Cardiovascular Risk and Nutrition Research Group (CARIN), Hospital del Mar Research Institute, Barcelona, Spain; 4 Gasol Foundation, Sant Boi de Llobregat, Barcelona, Spain; 5 Consortium for Biomedical Research in Epidemiology and Public Health, (CIBERESP), Instituto de Salud Carlos III, Madrid, Spain; 6 Maternal & Child University Hospital of Gran Canaria (HUMIGC), Canarian Health Service, Las Palmas de Gran Canaria, Spain

**Keywords:** educational factors, obesity, parity and maternal age, pregnancy, trends

## Abstract

**Introduction:**

The global prevalence of obesity has increasingly affected women of reproductive age, leading to several adverse outcomes during pregnancy, childbirth, and for their newborns. The objective of this study was to estimate time trends in early pregnancy among women who delivered live births in Gran Canaria (Spain) from 1993 to 2022 with stratified analysis by age, educational level, and parity.

**Methods:**

Data were obtained from 178,305 pregnant women who delivered at the Maternal & Child University Hospital of Gran Canaria (HUMIGC) between 1993 and 2022. The study period was divided into three intervals: 1993–2001, 2002–2010, and 2011–2022. Trends of early pregnancy obesity prevalence were examined globally and by educational level, age groups, and parity categories. Analysis was performed by multivariable logistic regression, adjusted for potential confounder variables.

**Results:**

The prevalence of obesity in early pregnancy increased significantly from 8.3% (95% CI 8.0–9.0) in 1993 to 22.5% (95% CI 21.0–24.0) in 2022. Multivariable logistic regression analysis adjusted for age, educational level, and parity revealed that obesity increased significantly in 2002–2010 (OR 1.73. 95% CI: 1.67–1.95) and further in 2011–2022 (OR 2.59, 95% CI 2.50–2.69) compared to 1993–2001. This upward trend was observed across all age groups, educational levels, and parity categories. Formal interaction analyses showed that the increase over time differed significantly according to age, educational level, and parity (all *p* for interaction < 0.001), with the greatest increases among women with university education and primiparous women.

**Conclusions:**

The prevalence of obesity during early pregnancy significantly increased in Gran Canaria (Spain) between 1993 and 2022. This increase was consistent across age groups, educational levels, and parity categories. Prenatal counselling should be implemented to help overweight women lose weight before pregnancy, ensuring they are fully informed about the potential risk of excess weight on pregnancy, childbirth, and neonatal outcomes.

## 1 Introduction

The global prevalence of obesity has increased markedly over recent decades. A pooled analysis of 3,663 population-based studies including 222 million participants estimated that, in 2022, obesity affected 13.1% of men and 15.1% among women worldwide (1). In Spain with data extracted from the complete historical series of the Spanish National Health Survey and the European Health Survey in Spain, the prevalence of obesity increased from 7.3% in 1987 to 15.7% in 2020 and this increasing trend has also been observed in women of reproductive age (2).

This is of special concern because maternal obesity during pregnancy increases the risk of obesity and metabolic disorders in offspring throughout life (3). Children born to obese mothers are more likely to develop overweight, type 2 diabetes, and cardiovascular issues due to fetal metabolic programming and epigenetic changes. Additionally, maternal obesity can influence infant growth patterns and neurodevelopment. Managing weight before and during pregnancy is crucial to reducing these adverse health outcomes and improving long-term well-being for both mother and child (3–9).

Importantly, obesity does not affect all women equally. The relationship between educational level and obesity is well established. Socioeconomic indicators such as educational level are strongly associated with obesity risk in women of reproductive age (10). On the other hand, several studies have found that there is a positive association between parity and overweight/obesity (11, 12). Furthermore, not all women living with obesity are at equal risk for pregnancy complications: maternal characteristics assessed for association with adverse outcomes in obese women included maternal age (13). The combined effect of Advanced Maternal Age and either overweight or obesity appeared to be a high-risk state particularly for stillbirth and preterm delivery (14).

Specifically, the patterns and changes in obesity prevalence among pregnant women across different age groups, parity categories, and educational backgrounds remain largely unexplored. Understanding these trends is crucial for identifying at-risk populations and informing targeted interventions.

Despite the fact that women in the first trimester of pregnancy represent a key target group for preventing excessive weight gain, highlighting the importance of preconception weight management and appropriate weight gain to mitigate associated risk to date, Spain still lacks national or regional population-based studies on monitoring secular trends of obesity in early pregnancy.

Therefore, this study aims to address this gap by examining obesity prevalence in early pregnancy over a 30-year period among women who delivered live-born infants in Gran Canaria, a Spanish community where obesity prevalence exceeds the national average (2). Additionally, we performed stratified analysis by age, educational level, and parity to identify specific subgroups of women who were at elevated risk of developing obesity during early pregnancy. To our knowledge, this is the first study conducted in Spain to analyse trends in maternal obesity over the past 30 years, and one of the few studies in Europe to examine differences in the evolution of these trends according to educational level. We hypothesized that obesity prevalence at the beginning of pregnancy has increased over time and that both the prevalence and temporal trends differ according to educational level.

## 2 Materials and methods

### 2.1 Study population

A repeated cross-sectional analysis of hospital-based registry data was conducted from 1993 to 2022 including 178,305 singleton pregnancies women who delivered live-born infants at HUMIGC. Data from 2013 was removed due to inconsistencies caused by changes in the data collection system, rendering them invalid for analysis. This cohort represents approximately 85% of all births at Gran Canaria during this period (15). A total of 2,927 participants (1.67% of the sample) were excluded due to missing or incorrect data regarding weight, height or age. Additionally, 761 women aged ≥50 years (0.4% of the sample) were excluded. The final sample included 174,617 participants, with complete data of BMI, age (and age groups) and parity. However, educational level data were available for 172,048 women.

Data on maternal characteristics were obtained from clinical records registered at the Gynecology and Obstetrics Service at HUMIGC.

### 2.2 Ethics approval

The present study was conducted in accordance with the guidelines set forth in the Declaration of Helsinki. The protocol was approved by the Human Research Ethical Committee of the Maternal & Child University Hospital of Gran Canaria (HUMIGC) on January 11, 2023 with the CEim code number 2023-015-1.

### 2.3 Anthropometric variables

Weight and height were measured during the first prenatal visit at the medical center, with participants wearing light clothes and no shoes. This visit most often takes place between gestational week 8–12. Overweight and obesity prevalence were assessed using the Body Mass Index (BMI) calculated as weight (kg)/ height^2^ (m^2^). BMI was categorized according to the Guidelines of American Clinics for the Identification, Evaluation, and Treatment of Obesity and Overweight in Adults: as follows: Underweight (BMI < 18.5 kg/m^2^), Normoweight (BMI 18.5–24.9 kg/m^2^), Overweight (BMI 25–29.9 kg/m^2^) and Obese (BMI ≥ 30 kg/m^2^) (16).

### 2.4 Other variables

Information on age, educational status and parity prior to pregnancy was collected. Age was categorized into three groups: <35 years, 35–39 years, and ≥40 years.

Educational level was determined based on the highest level of education attained and categorized as (i) low (primary school studies or no formal education), (ii) medium (secondary school education), and (iii) high (university education). Parity was classified into three categories: 1 child (parity 1), 2 children (parity 2), and ≥3 children (parity 3). The study period was divided into three intervals: 1993–2001, 2002–2010, and 2011–2022. The range of time periods was 9 years (1993–2001 group; *n* = 63,542 women) for the first and second period (2002–2010; *n* = 61,541 women), and 12 years for the final period (2011–2022 group; *n* = 49,534 women). The longer period for the latter group was chosen because birth rates declined after 2010; therefore, extending the time frame helped compensate for the lower number of pregnant women while approximating a decade for each group.

### 2.5 Statistical analysis

Descriptive statistical analysis included the calculation of means, standard deviations, and proportions of baseline characteristics across maternal study period categories. The association between study period categories and characteristics was analyzed by the Chi-squared test for proportions and the comparisons of absolute means between groups for age and BMI with ANOVA test.

Global secular trends in obesity in early pregnancy were assessed using multivariable logistic regression models, with obesity (yes/no) as the dependent variable and time period (1993–2001, 2002–2010, and 2011–2022) as the main independent variable.

The earliest period (1993–2001) was used as the reference category. The model was adjusted for maternal age, educational level, and parity. Additionally, to examine whether the temporal trend differed across population subgroups, stratified analyses were performed by age group (<35, 35–39, ≥40 years), educational level (primary, secondary, university), and parity (1, 2, ≥3 children). In the stratified models, estimates were adjusted for the remaining covariates (age and parity for educational level analyses; education and parity for age analyses; and age and education for parity analyses). This analysis includes 172,048 women with complete educational data. Finally, we repeated the logistic regression analysis using obesity prevalence as the dependent variable and year of examination as a continuous independent variable. Finally, to assess whether secular trends differed according to maternal age, educational level, and parity, separate multivariable logistic regression models including interaction terms between study period and each stratification variable were fitted. In addition, a logistic regression model including year of examination as a continuous variable was fitted to estimate the annual change in obesity prevalence. Statistical significance of the interaction terms was assessed using Wald tests. To evaluate the potential impact of the missing 2013 data, a sensitivity analysis was performed assuming obesity prevalence values similar to those observed in the adjacent years. Statistical analyses were conducted using IBM SPSS Software 23.0.

All *p*-values presented are two-tailed, and statistical significance was defined *a priori* at *p* < 0.05.

## 3 Results

### 3.1 Participant characteristics

The final sample included 174,617 women, with a mean age of 30.1 ± 6.3 years (range: 13–49 years). The mean maternal BMI at the start of pregnancy was 24.9 ± 5.1 kg/m^2^ (range: 13.2–61.7).

The main characteristics of the participants according to the time periods are presented in Table 1. The overall age-adjusted prevalence of overweight and obesity was 25.1% and 15.0%, respectively. Obesity prevalence was higher among oldest women, from low educational backgrounds, and multiparous. Mean maternal age and BMI rose over the three study periods. Fewer women were under 35, while those aged 40 or older increased significantly (*p* < 0.0001). Maternal education levels also grew, with fewer women having low education and more attaining university degrees (*p* < 0.0001). Parity changed minimally, but there was a slight rise in women with three or more children in the latest period.

**Table 1:** Particpant characteristics by time period.

		Time period				
		1993–2001	2002–2010	2011–2022	Total	*p* value
		*n* = 63,542	*n* = 61,541	*n* = 49,534	*n* = 1,74,617	
Age	Mean ± sd	28.3 ± 5.6	29.6 ± 5.9	33.2 ± 6.4	30.1 ± 6.3	<0.001*
	<35 (%)	86.2	77.6	56.7	74.8	<0.001**
	35–39 (%)	11.8	18.7	26.2	18.4	–
	≥40 (%)	2.0	3.6	17.1	6.9	–
Educational level	Primary school (%)	72.5	53.0	33.8	54.9	<0.001**
	Secondary school (%)	15.0	29.1	34.8	25.5	–
	University (%)	12.5	17.9	31.4	19.7	–
Parity	Parity 1 (%)	41.9	42.9	40.0	41.7	<0.001**
	Parity 2 (%)	33.3	32.7	32.4	32.8	–
	Parity ≥ 3 (%)	24.9	24.3	27.6	25.5	–
BMI	Mean ± sd	24.0 ± 4.5	25.0 ± 5.1	25.9 ± 5.6	24.9 ± 5.1	<0.001*
	Underweight (%)	5.5	4.3	3.9	4.6	<0.001**
	Normoweight (%)	60.9	54.9	48.2	55.2	–
	Overweight (%)	23.2	25.3	27.3	25.1	–
	Obesity (%)	10.3	15.5	20.5	15.0	–

s.d = standard deviation; BMI = body mass index.

* ANOVA test.

** Chi-squared test. The educational level includes only 172,048 women.

In the multivariable analysis (Table 2), adjusting for educational level, age groups and parity the odds of early pregnancy obesity compared to women in 1993–2001, increased from 1.73 (95% confidence interval: 1.67–1.79) in 2002–2010 to 2.59 (95% confidence interval: 2.50–2.69) in 2011–2022. A significant increase in early pregnancy obesity (*p* < 0.001) was observed across all age groups, educational levels, and parity categories (Table 2). However, this trend was somewhat stronger among mothers with medium to higher education, as well as among first-time mothers, in comparison to those with lower educational attainment or more than 1 child, respectively.

**Table 2:** Logistic regression analysis of secular trends of obesity in early pregnancy according to age, educational level, and parity.

	*n*	1993 to 2001 (*n* = 63324)	2002 to 2010 (*n* = 61,076)			2011 to 2022 (*n* = 47,648)			*P* for trend	*P* for interaction
			OR	95% CI	*p*	OR	95% CI	*p*		
All*^,^^a^	1,72,048	1 (reference)	1.72	1.66–1.78	<0.001	2.49	2.40–2.2.58	<0.0001	<0.001	–
Age groups^b^	–	–	–	–	–	–	–	–	–	<0.001
<35 years	1,29,007	1 (reference)	1.77	1.71–1.84	<0.001	2.56	2.46–2.68	<0.001	<0.001	–
35–39 years	31,432	1 (reference)	1.60	1.47–1.74	<0.001	2.52	2.32–2.75	<0.001	<0.001	–
≥40 years	11,609	1 (reference)	1.22	1.02–1.46	0.032	2.18	1.85–2.55	<0.001	<0.001	–
Educational level^c^	–	–	–	–	–	–	–	–	–	<0.001
Primary school	94,398	1 (reference)	1.60	1.54–1.67	<0.001	2.11	2.02–2.21	<0.001	<0.001	–
Secondary school	43,804	1 (reference)	2.09	1.92–2.29	<0.001	3.22	2.95–3.51	<0.001	<0.001	–
University	33,846	1 (reference)	2.10	1.84–2.39	<0.001	3.50	3.09–3.96	<0.001	<0.001	–
Parity^d^	–	–	–	–	–	–	–	–	–	<0.001
Parity 1	71,837	1 (reference)	1.95	1.80–2.02	<0.001	2.76	2.59–2.94	<0.001	<0.001	–
Parity 2	56,467	1 (reference)	1.73	1.63–1.83	<0.001	2.55	2.39–2.72	<0.001	<0.001	–
Parity ≥ 3	43,744	1 (reference)	1.54	1.49–1.64	<0.001	2.21	2.07–2.35	<0.001	<0.001	–

* OR: odds ratio; CI: confidence interval.

a Cross-sectional studies from 1993 to 2001 as reference group adjusted for educational level. age and parity.

b adjusted for education level and parity.

c adjusted for age and parity.

d adjusted for age and educational level.

This analysis included only women with available educational level data (*n* = 172,048). *P* values correspond to comparisons with the reference period (1993–2001). *P* for interaction was obtained from multivariable logistic regression models including interaction terms between study period and age group, educational level, or parity, respectively. *P* for trend was obtained by modelling study period as an ordinal continuous variable in the multivariable logistic regression models.

Logistic regression analysis with the prevalence of obesity as the outcome and year of examination (continuous) as the exposure showed a 4.7% annual increase in the odds of obesity [OR: 1.047 (95% CI: 1.045–1.049)]. Formal interaction analyses further demonstrated that secular trends differed significantly according to maternal age, educational level, and parity (all *p* for interaction < 0.001). Specifically, the increase in obesity prevalence over time was significantly greater among women with secondary and university education than among those with primary education, and among primiparous women compared with multiparous women. Conversely, women aged ≥40 years experienced a significantly smaller increase over time than women aged <35 years.

A sensitivity analysis assuming obesity prevalence values for 2013 similar to those observed in adjacent years showed an identical yearly increase in obesity prevalence compared with the analysis excluding the missing year.

### 3.2 Changes over time

**Figure 1:**
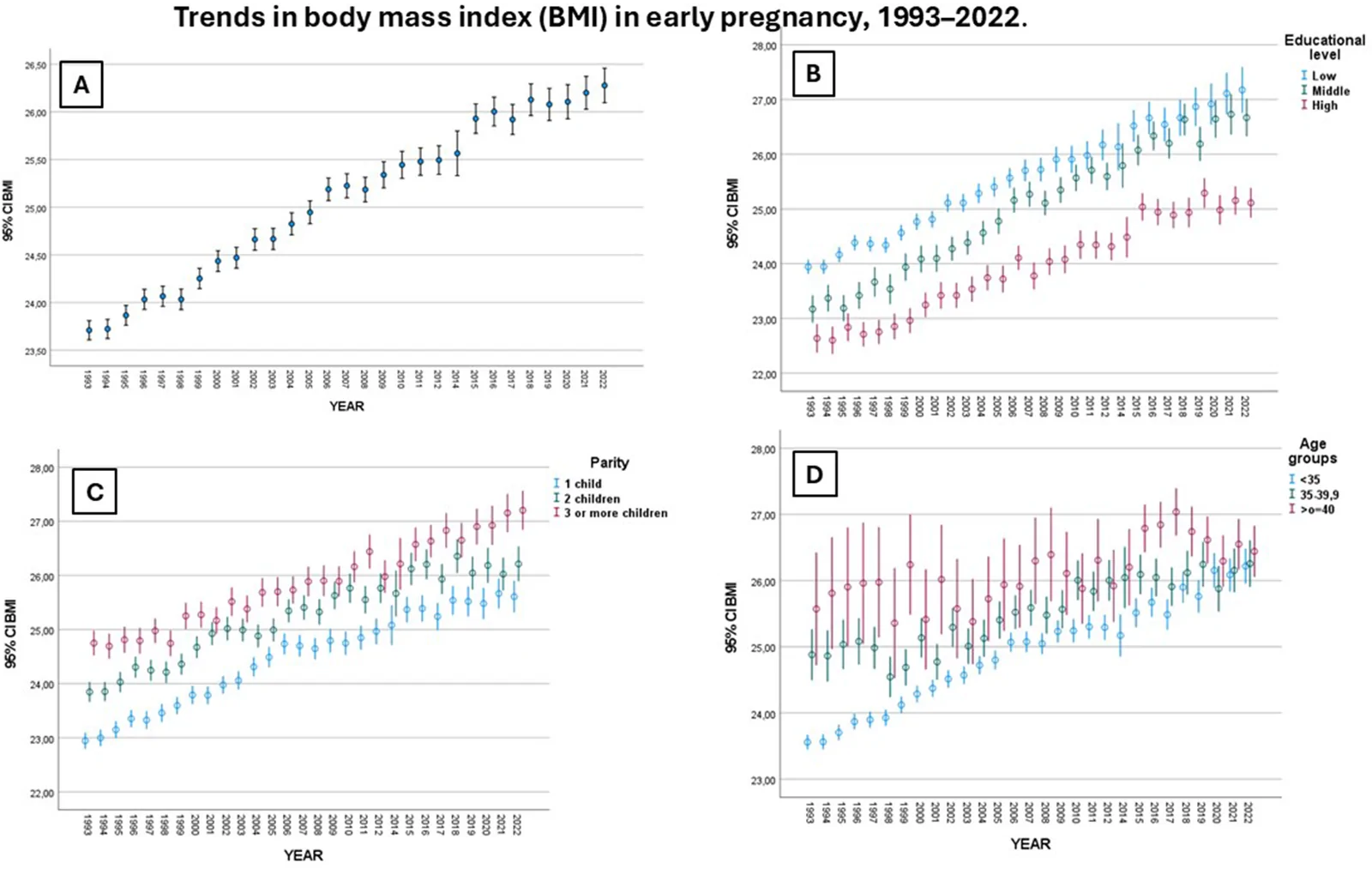
Trends in body mass index (BMI) in early pregnancy, 1993–2022.

**Figure 2:**
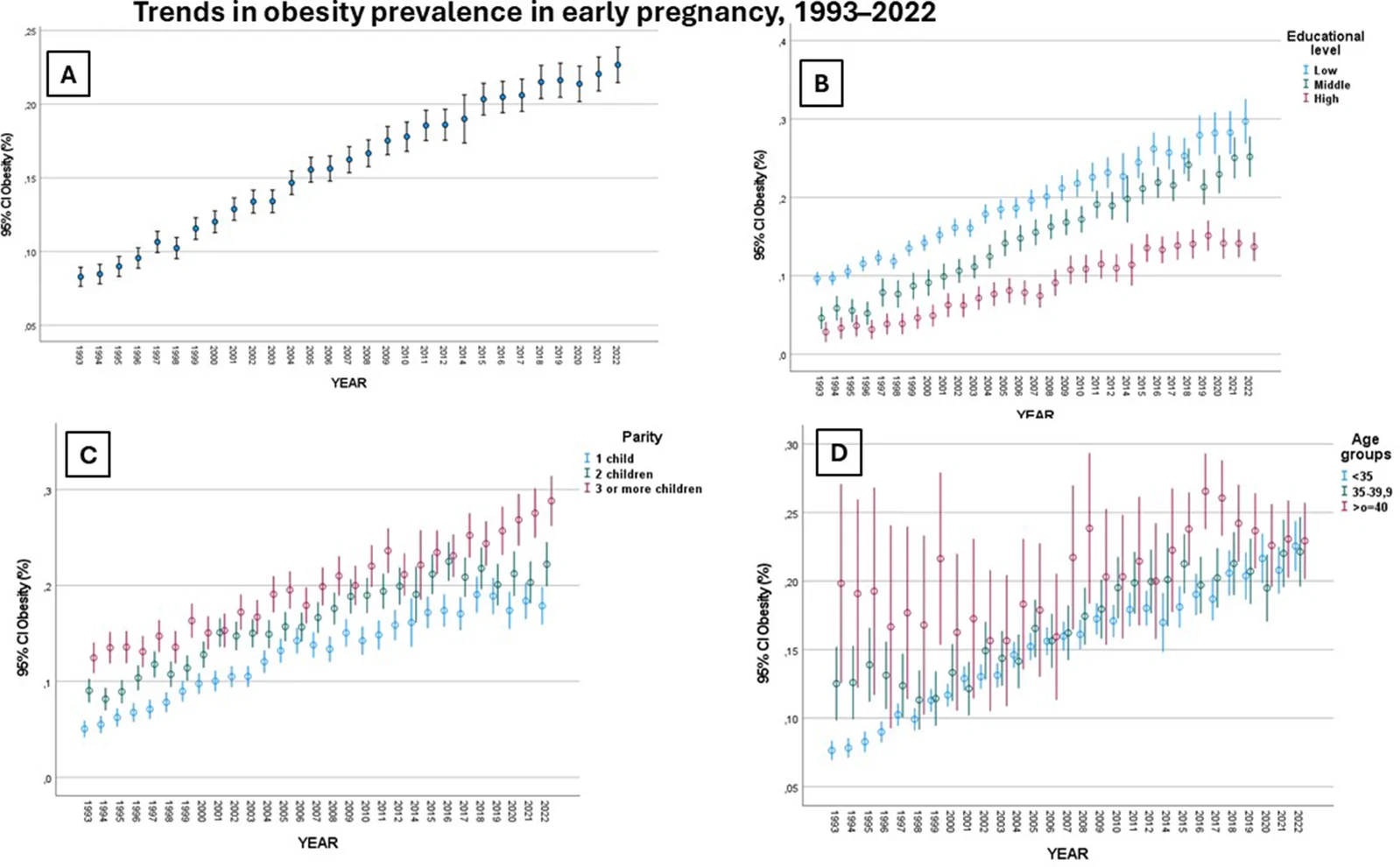
Trends in obesity prevalence in early pregnancy, 1993–2022.

BMI and obesity prevalence evolution:

Between 1993 and 2022, the mean age-adjusted mean BMI increased from 23.7 (95% CI: 23.6–23.8) in 1993 to 26.2 (95% CI: 26.0–26.4)). Obesity prevalence increased from 8.3% (95% CI 8.0–9.0) in 1993 to 22.5% (95% CI 21.0–24.0) in 2022, respectively. An increase in BMI and obesity prevalence were observed across all educational levels, parity groups and age groups (Figures 1,2).

## 4 Discussion

The main findings of this study were that the prevalence of obesity at the beginning of pregnancy increased markedly between 1993 and 2022 and that this increase was observed across all maternal age groups, educational levels, and parity categories. Formal interaction analyses further demonstrated that the magnitude of this increase differed significantly according to these characteristics, with greater increases among women with secondary and university education and primiparous women, whereas women aged ≥40 years experienced a smaller increase over time than younger women.

The rising prevalence of pre-pregnancy obesity is particularly alarming given the well documented dose–response relationship between increasing pre-pregnancy BMI and the elevated risk of obstetric (17). In a previous study conducted on 6,558 women in early pregnancy (a subsample from the present study population), we found that compared to normal weight women, obese women had significantly higher risks of developing gestational diabetes mellitus, gestational hypertension, preeclampsia, oligodramnios, polyhydramnios, perineal tearing, and were more likely to undergo caesarean section and manual placenta extraction. Additionally, newborns of overweight and obese women were found to have higher birth weights and an increased risk of macrosomia, as well as a greater likelihood of requiring admission to special care units. Moreover, their Apgar scores at 1 min were significantly lower (17). Finally, some studies have found that not only higher maternal early pregnancy BMI has influence in large weight for gestational age (18), but are associated with an increased risk of childhood overweight/obesity, with the strongest effects at later ages (19).

Our results are consistent with those reported in two recent systematic reviews and meta-analyses conducted by Martínez-Hortelano in 2020 (20) and Kent in 2024 (21) on global trends in obesity prevalence over time. These reports identified a significant increase in maternal obesity prevalence worldwide. Specifically, Kent et al., using data from 1980 to 2020, reported a significant rise in maternal obesity prevalence over time. They observed the greatest increases in North America, Australia, and Oceania, whereas the lowest increases were in Asia. The authors also estimated the current global prevalence of maternal obesity at 20.9% and projected that it will increase to 23.3% by 2030. more, they reported a yearly increase in obesity risk of 0.34%, whereas in the present study we observed an annual increase of 4.7%.

Similarly, in the study by Martínez-Hortelano et al., the global prevalence of prepregnancy overweight and obesity during the period 2009–2018 was estimated at 16.3%. The highest prepregnancy prevalence rates were reported in North America, whereas the lowest were observed in Asia. Both studies highlighted that most available evidence originated from Europe and the Americas, while data from other regions, particularly Africa and South America, were limited. Consequently, comparing temporal trends in obesity at the start of pregnancy between developed and developing countries remains very complicated because in many low- and middle-income countries, health information systems are incomplete or unavailable (22).

To our knowledge, there are no national or regional studies in Spain on the evolution of the prevalence of obesity at the start of pregnancy. Regarding prevalence studies in Spain, only two hospital-based studies were identified that report on the prevalence at the start of pregnancy, both using self-reported anthropometric data, and neither assessed temporal trends. The study by Melchor I et al. (23), conducted in a hospital in Vizcaya (2013–2017), showed an obesity prevalence of 13.3%. During the same period, we obtained a prevalence of 20.3%. In the study by González-Plaza E et al. (24), conducted in a hospital in Barcelona during the period 2015–2016, showed an obesity prevalence of 8.4%. During the same period (2015–2016), we obtained much higher obesity prevalence, at 20.5%. The data show that obesity is less prevalent in these regions of Spain than in the Canary Islands, where the studied hospitals are located. Since the figures are self-reported, the prevalence may be underestimated. Our findings show a marked increase in obesity prevalence at the start of pregnancy, consistent with trends reported globally, across Europe, in Spain, and in the Canary Islands. Although most population estimates are based on self-reported data, obesity trends among pregnant women appear to reflect those of the general female population in the same region (1, 2). In Spain, analyses of the Spanish National Health Survey and the European Health Survey (1987–2020) showed that obesity prevalence in women increased until 2001 and then stabilized, except among women aged 15–24 years, in whom it continued to rise. A similar pattern has been reported in the Canary Islands, where obesity prevalence increased by more than 150% between 1987 and 2020 (2). Consistent with these data, obesity prevalence at the start of pregnancy increased by 171% in our cohort between 1993 and 2022.

One of the findings of the present study underscores the influence of educational level on obesity prevalence at the beginning of pregnancy. Women with a high educational level had a lower risk of obesity compared to those from low educational backgrounds throughout the study period. These findings are consistent with epidemiological studies that have reported a progressive increase in obesity prevalence among pregnant women over recent decades, with a greater risk of obesity among women with lower educational levels (25–27).

However, the results of the present study also show that the increase in obesity prevalence over time was greater among women with medium and high education levels compared with those with lower educational levels. In accordance with this pattern, multivariate logistic regression analysis showed a larger relative increase in early pregnant obesity among women with secondary and university education compared to those with primary education. Importantly, groups of medium and high education started from substantially lower baseline levels of obesity. These findings indicate that the temporal increase in obesity prevalence differed according to educational level, with relatively greater increases among women with secondary and university education despite their lower baseline prevalence. Although the obesogenic environment is strongly present in disadvantage areas (28) it has also affected social groups traditionally protected by higher socioeconomic status (29). Consequently, the protective effect of higher education may be weakening, leading to faster increases in obesity in these groups. It should be noted that the results obtained may have been influenced by the fact that access to higher education in Spain has undergone drastic changes over the past three decades and that the socioeconomic implications of university education may not have been the same in 1993 as in 2022.

In contrast to our findings, the study conducted by Bjermo et al. which analyzed data from the Swedish Medical Birth Register between 1992 and 2010, including 1,569,173 singleton pregnancies, reported that the prevalence of obesity during pregnancy increased significantly in Sweden over this period, along widening social inequalities in obesity. The odds of obesity among women with lower educated compared to women to those with higher education increased from 1.91 (95% CI: 1.85–1.97) in 1992–1995 to 2.09 (95% CI: 2.05–2.14) in 2008–2010 (26). Similarly, the study published by Vogt et al. in 2024 (27), based on cross-sectional data from a repeated survey in Vasterbotten, Sweden, included 18,568 pregnant women and 18,110 male partners between 2010 and 2019. The study concluded that obesity prevalence among women increased from 9.4% in 2010 to 11.7% in 2019, while educational inequalities in obesity remained consistent throughout the study period. The analysis of different time periods, the differing classifications of socioeconomic levels, the lack of adjustment for key confounding variables such as parity and age groups, and the fact that the data were mainly self-reported make the findings difficult to compare with those obtained in the present study.

In relation with parity and age, we found that the strongest increase within strata was found in women aged less than 35 years and primiparous women. These results are different from those obtained by Strauss et al. (30), that analyzed 335,511 mothers in the Federal State of Schleswig-Holstein (German Perinatal Survey) and examined the dynamics of maternal anthropometric variables over more than two decades (1995–2017) in Germany, they found that the proportion of obese women increased disproportionately, with an average rise from 9.4 to 19.2%, and that it was not advanced maternal age but parity that influenced the continuous increase in maternal weight. Our results are similar to Straus's regarding the influence of age-related obesity trends, because we found that the increase was greater in women under 35 than in those over 35 and 39 years. Although older age and the higher proportion of women of advanced maternal age (AMA) could be related to the increase in pregnant obesity, our results do not fully explain this relationship. A summary of studies yielding results similar to ours can be found in Supplementary Table S1.

Several factors may explain the increasing prevalence of obesity at the beginning of pregnancy observed over recent decades. These include the progressive rise in obesity among women of reproductive age (1), changes in dietary habits and physical activity patterns (31), and lifestyle changes associated with increasingly obesogenic environments (32, 33).

Finally, the results of this study highlight the need for early interventions targeting women who are planning a pregnancy or who begin pregnancy with overweight or obesity, with the aim of reducing pregnancy-related risks. In the meta-analysis conducted by Thangaratinam in 2012 on the effects of interventions during pregnancy on maternal weight, the authors concluded that dietary and lifestyle interventions during pregnancy can reduce gestational weight gain and improve outcomes for both the mother and the infant. Among the different strategies evaluated, diet-based interventions were found to be the most effective, being associated with reductions in maternal gestational weight gain as well as improved obstetric outcomes (34).

### 4.1 Strengths and limitations

To our knowledge, this is the first study conducted in the Spanish population to examine the long-term evolution of weight status among women during early pregnancy. The extensive study period, high sample size and the fact that the data were measured rather than self-reported and compiled within a single database, enhance the study's reliability and scientific value, further supporting the relevance of the findings.

One of the limitations of the present study is that it is not representative of the Spanish pregnant population; therefore, the results cannot be extrapolated to the general Spanish pregnant population.

Another limitation of this study is that we do not have data on the variable “assisted reproductive technology”, the use of which has increased notably in recent years, and which could be associated with both advanced maternal age and variations in weight gain.

A further limitation is that it only includes pregnant women attending a public hospital in Gran Canaria. However, this population accounted for approximately 85% of all births in Gran Canaria during the study period, supporting the representativeness of the findings at the regional level. Nevertheless, we cannot exclude the possibility that women delivering outside the public hospital system may differ in some socioeconomic characteristics from those included in the present study. Therefore, the possible bias related to socioeconomic status should be taken into consideration (15). Additionally, data from 2013 was excluded from the analysis due to a transition period in the hospital's data collection system, following a change in the hospital's software program. As a result, the completeness of the sample of pregnant women from that year could not be ensured, necessitating their exclusion from the study. Furthermore, the objective of the present study was to analyse long-term trends rather than year-to-year variability. The absence of data for one year in this long time series is minimal (3.3% of the study period) and unlikely to have significantly affected the results.

## 5 Conclusions

The odds of obesity during early pregnancy increased significantly in Gran Canaria (Spain) between 1993 and 2022 independently of age, educational level, and parity. The temporal increase in obesity prevalence was greatest among primiparous women and women with secondary or university education, whereas women aged ≥40 years experienced a smaller increase over time than younger women.

It is crucial to emphasize that these adverse effects do not end with delivery or the postpartum period, but instead represent a livelong burden for both mothers and their offspring, further perpetuating the intergenerational cycle of obesity.

Since pregnancy is a critical period during which motivation for adopting risk-reducing behaviours may be higher, antenatal care could serve as an optimal setting for implementing preventive interventions. Effective primary and clinical interventions should be integrated into primary care and antenatal care settings to help mitigate these risks. Public health policies should take into account the importance of preventing obesity at the beginning of pregnancy and, in clinical settings, address the problem early through effective dietary and lifestyle interventions in overweight pregnant women (34).

## Data Availability

The raw data supporting the conclusions of this article will be made available by the authors, without undue reservation.
