# Supplementary material for: Trends in obesity prevalence during early pregnancy in Gran Canaria, Spain, 1993 to 2022
Source: Front Reprod Health. 2026 Aug 26;8:1922881. doi: 10.3389/frph.2026.1922881 (PMC13553775; doi:10.3389/frph.2026.1922881)
Supplement: Supplementary file 1 [file Datasheet1.pdf]

Supplementary table 1. Overview of comparable studies

**Study**	**Time period**	**Countrywide or regional**	**Design**	**Obesity diagnostic**	**Subjects (n)**	**Obesity prevalence**	**Sociocultural factors: different prevalences**	**Sociocultural factors: different trends**
Bautista et al.	1993-2022	Regional (Spain, Gran Canaria)	Repeated cross-sectional study	Direct measurement of maternal height and weight recorded at the first pregnancy visit.	174,617	8.3 to 22.5%	More obesity prevalence in low educational level	Increase in obesity risk of higer and middle educated vs lower from 1993-2001 to 2002-2010 and to 2011-2022
Melchor(23) (2019) Spain	2013-2017	Regional (Spain,Vizcaya)	Cross-sectional study	Self-reported prepregnancy weight and height	16,609	13.3%	No	No
González-Plaza(24) (2021) Spain	2015-2016	Regional (Spain, Barcelona)	Cross-sectional study	Self-reported prepregnancy weight and height	5,447	8.4%	No	No
Heslehurst (25)(2007) UK	1990-2004	Regional (Middlesbrough ,UK)	Repeated cross-sectional study	Direct measurement of maternal height and weight recorded at the first pregnancy visit. (only 5% self-reported)	36,821	9.9 to 16.0%	More obesity prevalence in more deprivation áreas	No

Bjermo(26)(2005) Sweden	1992-2010	National (Swedish Medical Birth Register)	Repeated cross-sectional study	Most weights were measured, during the first visit at the antenatal-care clinic. (some weights self-reported)	1,569,173	5.1 to 9.6%	More obesity prevalence in low educational level	Increase in obesity risk of lower educated vs higher from 1992–1995 to 2008–2010
Strauss, (30)2021 Germany	1995-97 and 2004-2017	Regional (Federal state of Schleswig-Holstein, Germany)	Repeated cross-sectional study	Recorded for National Perinatal Survey of Quality Indicators	335,511	9.4 to19.2%	No	No
Vogt (27) (2024) Sweden	2010-2019	Regional (Västerbotten, Sweden)	Repeated cross-sectional study	Self-reported prepregnancy weight and height	18,568	9.4 to 11.7%	More obesity prevalence in low educational level (2019)	Educational inequalities in obesity remained consistent throughout the study period
